# Misreporting of height and weight by primary school children in Japan: a cross-sectional study on individual and environmental determinants

**DOI:** 10.1186/s12889-023-15682-z

**Published:** 2023-04-27

**Authors:** Sachie Mori, Keiko Asakura, Satoshi Sasaki, Yuji Nishiwaki

**Affiliations:** 1 Department of Environmental and Occupational Health, Toho University Graduate School of Medicine, Omori-Nishi 5-21-16, Ota-Ku, Tokyo, #143-8540 Japan; 2 Department of Environmental and Occupational Health, School of Medicine, Toho University, Omori-Nishi 5-21-16, Ota-Ku, Tokyo, #143-8540 Japan; 3 Department of Social and Preventive Epidemiology, School of Public Health, the University of Tokyo, Hongo 7-3-1, Bunkyo-Ku, Tokyo, #113-0033 Japan

**Keywords:** Misreporting, Height, Weight, BMI z-score, Body size perception, Primary school children, Japan

## Abstract

**Background:**

Appropriate body constitution during childhood is important for future health. However, it has been suggested that thinness is increasing among adolescent girls and boys in Japan. Since misreporting of height/weight may be a possible reflection of the child's ideal body image, we investigated the magnitude and direction of height/weight misreporting and its determinants among Japanese young adolescents.

**Methods:**

A total of 1019 children in public primary schools were included in the analysis. Both measured and self-reported values of height/weight were obtained. Misreporting of height/weight was calculated by subtracting the measured value from the self-reported value. The association between misreporting and several variables such as the BMI z-score of individuals and body constitution of surrounding children was explored by multivariate linear mixed models.

**Results:**

As BMI z-score increased, ‘overreporting’ of height by boys and ‘underreporting’ of height by girls became larger (*p* = 0.06 in boys, *p* = 0.02 in girls). Both boys and girls with a larger body size tended to underreport their weight (*p* < 0.01 in boys, *p* < 0.01 in girls). Boys who belonged to a school with a larger average BMI z-score were more likely to overreport their weight. This tendency was not observed for girls.

**Conclusions:**

Self-reported height/weight was generally accurate in Japanese primary school children. However, even primary school children misreported their height/weight intentionally like adults, possibly due to social pressure to lose weight or that not to stand out. Thus, health education about appropriate body constitution should be provided from the beginning of adolescence, particularly for girls.

**Supplementary Information:**

The online version contains supplementary material available at 10.1186/s12889-023-15682-z.

## Background

Maintenance of an appropriate body constitution is important to reducing the risk of non-communicable diseases [1] and mortality [2]. The relationship between body constitution and mortality is known to follow a U-shaped curve, meaning that not only obesity but also thinness increase mortality in adults [3]. An appropriate range of body constitution differs by race, age, and sex [4]. The proportion of obese adults in Japan is relatively low (4% to 5%) [5] compared to Western countries, but the proportion of thin women in their twenties reaches as high as 20% [6]. Thinness in young women can cause menstrual disorders [7] and decreased bone density [8]. This issue of thinness has also been observed among adolescent girls in Japan [9, 10], and the proportion of thinness has been reported to have increased among adolescent boys [11].

While height and weight are objectively measured using scales in health check-ups, large-scale epidemiological studies often use self-reported height/weight, and are accordingly subject to the problem of misreporting. The problem of self-reporting is further complicated by a range of factors associated with the magnitude and direction of body constitution misreporting: in general, many studies have reported that overweight/obese people underreport their weight [12, 13], or are unaware of their overweight [14]. Misreporting may also be associated or attributable to social norms which consider that obesity is unfavorable, based on the common notion that overweight/obesity reflects from a lack of self-discipline and individual responsibility [15]. In addition, a survey of Japanese aged reported that overreporting of body mass index (BMI) is greater in people with a short period of education [16], while another found that the degree of misreporting of body constitution was smaller in Asian than similarly matched Western populations [13]. Other studies report that obesity was hardly realized when more overweight people were present [17, 18]; thus, environmental factors may affect the awareness of one’s weight.

Of note, most of these studies were conducted in adults [13], and few studies in children have yet appeared. Moreover, most focused mainly on the misclassification of obesity [19]. To our knowledge, the misreporting of body constitution and its determinants in Japanese children has not been investigated.

Here, we investigated the magnitude and direction of height/weight misreporting among Japanese primary school children. We also explored variables which affect their misreporting, from both individual and environmental perspectives, and considered whether causes of misreporting among the children differed from those in adults.

## Methods

### Participants

The present study used the baseline data of a previous nutrition education intervention study, "Diet survey for children and their family." The design of that study has been described in detail elsewhere [20]. In brief, the survey was conducted in 14 public primary schools in a single prefecture in the Kanto area, in the central part of the main island of Japan, in May, 2018. All 5^th^ and 6^th^ graders enrolled in 14 primary schools were recruited. No exclusion criteria were set, because all children attending public schools in Japan are required to be educated equally. The 1,231 children in 7 of these 14 schools self-reported their height/weight, and were included in the present study. All children in the other seven schools, who did not self-report their height/weight, were excluded.

### Measurement of height and weight

Measurement of height/weight was conducted as part of a routine health check-up by school nurses at each school in April 2018. By law, measurements are conducted between April and June in Japan. Body height/weight was measured to the nearest 0.1cm and 0.1kg, respectively, with the child wearing light clothing and no socks. Body height was measured in an upright position with both heels close together; back, buttocks, and heels touching the scale post of the stadiometer; both arms hanging down; and the head in the eyes-ears horizontal position. Weight scales for the health check-up are mandatorily inspected once every two years according to Measurement Act [21], and the measured values are considered accurate. After the health check-up, the results were notified to the children and their guardians within 21 days. For the present study, the measured height/weight data were provided by the municipal boards of education to the researchers.

### Questionnaires

#### Distribution

Two questionnaires were used in this study. They were distributed and collected in May, 2018. The first was a brief-type self-administered diet history questionnaire (BDHQ15y) that was distributed at school, and filled out at home by the children and/or the guardians. The second questionnaire was a lifestyle questionnaire that asked about basic characteristics of children. It was distributed at school, and filled out at school by the children.

#### Information on self-reported height and weight

The BDHQ15y was developed for school children based on the BDHQ, which was designed to quantify food and nutrient intakes in Japanese adults over the preceding month [22, 23]. In the present study, information about body height, weight, and date of birth were collected using the BDHQ15y in May, 2018, i.e. after the health check-up at school in April. As described above, the children and/or their guardians in the seven schools self-reported the children’s height/weight to the first decimal place in cm and kg, i.e. to the nearest 0.1 cm and 0.1 kg, respectively. (In the other seven schools, measured height/weight was pre-entered in the BDHQ15y prior to self-administration.) Age in days was calculated as the duration between the date of birth written in the BDHQ15y and May 15, 2018 (date of questionnaire collection). The participants also answered who filled out the questionnaire in the BDHQ15y. The respondents were able to choose multiple items from the five choices of "child themself", "mother", "father", "grandmother", and "others". These were then further categorized as "child only" for those who chose only "child themself"; "guardian only" for those who did not choose "child themself"; and "child + guardian" for those who chose any combination of "child themself" and other choices.

#### Lifestyle questionnaire

The children answered questions about sex, self-awareness of weight ("Compared to other children in your class, do you think your weight is adequate, heavy, or light?"), intention to lose weight (answer: yes/no), nutrition knowledge, and frequency of exercise. The lifestyle questionnaire included a validated component on questionnaire on children’s nutrition knowledge [24]. This questionnaire included 27 questions such as knowledge about nutrients of foods, functions of nutrients, and the relationship between nutrients and health. The percentage (%) of correct answers was calculated. The frequency of exercise was defined as follows. The children were first asked "How many days do you exercise for 60 min or more in your usual week?” with responses from the eight choices of "0 days"-"7 days". These were then further categorized as "0 days"; "1 ~ 2 days" for "1 day" or "2 days"; "3 ~ 4 days" for "3 days" or "4 days "; and "5 ~ 7 days" for "5 days", "6 days" or "7 days".

### Statistical analysis

Among the 1231 enrolled children, we excluded those who did not provide consent to participate or did not give sufficient information through the questionnaire (*n* = 211) and those without measured height/weight (*n* = 1). Finally, 1019 children were included in the analysis (participation rate: 1019/1231*100 = 82.8%).

Misreporting of height/weight was calculated by subtracting the measured value from the self-reported value (measured and reported as mentioned above for both), and defined as overreporting of >0 or underreporting of <0. BMI was calculated as body weight divided by the square of body height (kg/m^2^) using the measured values.

First, the value of self-reported and measured height/weight as well as the magnitude of misreporting were described. Bland-Altman plot was depicted to examine the agreement of self-reported and measured height/weight and to check systematic difference between the measurements as well as outliers [25]. Then, Pearson’s correlation coefficient between self-reported and measured height/weight was calculated by sex. In children, assessing the appropriateness of body constitution by BMI itself is difficult because the appropriate BMI range fluctuates with growth. We therefore adopted a BMI standard curve which had been developed using the LMS method [26, 27]. We used this curve to calculate the SD score (BMI z-score), based on a formula developed using body constitution data of Japanese children in 2000. The number of overweight and underweight children was calculated based on the cutoff values established by Cole TJ et al. [28–30].

Next, the relationship between misreporting of height/weight and its possible determinants was analyzed using the t-test and analysis of variance (ANOVA) by sex. Trends of association were examined for ordinary variables using linear regression models which assigned scores to the level of the independent variables (e.g. small=0, medium=1, large=2). The variables following were considered: age, school, measured height/weight (categorized in tertiles), self-awareness of weight, intention to lose weight, nutrition knowledge level (categorized in tertiles), exercise frequency per week, and respondent to the questionnaire. To consider different grades of differences in body size, tertiles of height/weight for each grade were calculated separately, then integrated. The smallest group was defined as "small", the medium group as "medium", and the largest group as "large".

The relationship between misreporting of height/weight and BMI z-score as an index of body constitution was then examined using multivariate linear mixed models by sex. In this analysis, misreporting of height/weight was a dependent variable, and BMI z-score of individual children was a major independent variable, with schools as random effects. In Model 1, the BMI z-score of individuals was included as an independent variable. In Model 2, mean BMI z-score in each school was added to evaluate the effect of body constitution of children surrounding a certain child. Model 3 further included questionnaire respondent as an additional covariate. Self-awareness of weight and intention to lose weight were not included in the models irrespective of the results of the univariate analysis, because these variables were strongly related with the measured height/weight.

Lastly, to evaluate effect modification by sex on the relationship between misreporting of height/weight and actual body constitution, an interaction term (BMI z-score x sex) was included in multivariate linear mixed models. In this analysis, misreporting of height/weight was a dependent variable, and BMI z-score, sex, and the interaction term described above were independent variables, with schools as random effects. This analysis was performed for boys and girls together.

All analyses were performed with Stata/SE 15.1 for Windows (Stata Corp LLC, Texas, USA). Statistical tests were two-sided, and *p* values of < 0.05 were considered statistically significant.

## Results

Characteristics of the children are shown in Table 1. Average misreporting of height was -0.13 cm in boys and 0.09 cm in girls, while that of weight was -0.09 kg and -0.14 kg, respectively. Bland–Altman plot showed that the most of the values were within the limits of agreement (Supplementary Fig. 1 (boys), 2 (girls)). Pearson’s correlation coefficients for self-reported and measured values were close to 1.

**Table 1:** Characteristics of participants (*n* = 1019)

Sex	Variables	Self-reported		Measured^a^		Misreporting^b^				*P* value^c^	Correlation coefficient^d^
		mean,SD or n(%)				mean,	SD	min	max		
Boys (*n* = 486)	Age (year)	10.6,	0.6								
	Height (cm)	141.9,	7.8	142.0,	7.5	-0.13,	2.3	-12.3	9.3	0.097	0.96
	Weight (kg)	36.2,	8.2	36.3,	8.5	-0.09,	1.7	-10.7	10.0	0.114	0.98
	BMI (kg/m^2^)	17.9,	3.0	17.9,	3.1	0.01,	1.0	-5.7	4.6	0.456	0.95
	Body constitution^e^										
	Underweight	52	(10.7)	60	(12.3)						
	Normal weight	365	(75.1)	357	(73.5)						
	Overweight	69	(14.2)	69	(14.2)						
Girls (*n* = 533)	Age (year)	10.6,	0.6								
	Height (cm)	143.4,	7.6	143.3,	7.6	0.09,	1.5	-6.6	18.6	0.078	0.98
	Weight (kg)	36.2,	7.7	36.4,	8.1	-0.14,	2.0	-14.5	11.9	0.046	0.97
	BMI (kg/m^2^)	17.5,	2.5	17.5,	2.7	-0.07,	1.0	-6.3	6.2	0.039	0.94
	Body constitution^e^										
	Underweight	80	(15.0)	71	(13.3)						
	Normal weight	404	(75.8)	405	(76.0)						
	Overweight	49	(9.2)	57	(10.7)						

*BMI* body mass index, *SD* standard deviation

^a^ The "measured" values were regarded as the gold standard

^b^ Misreporting was defined as the difference between self-reported values and measured values (Misreporting = self-reported values—measured values)

^c^ Comparison between misreporting and zero value using t test

^d^ Pearson’s correlation coefficient between self-reported values and measured values was calculated

^e^ The cutoff values for underweight and overweight were < BMI18.5 and ≥ BMI25 at age 18, respectively

Relationships between misreporting of height/weight and possible determinants are shown in Table 2. In girls only, lighter measured weight (*p* = 0.02) and self-awareness that one’s weight is lighter than others (*p* < 0.01) were significantly associated with overreporting of height. For weight, the larger the measured height/weight, the larger the underreporting of weight (*p* = 0.04 for boys’ height, *p* < 0.01 for boys’ weight, *p* < 0.01 for girls’ height/weight). Also, self-awareness of weight was significantly associated with misreporting of weight (*p* < 0.01 for boys and girls). When the children recognized that their weight was lighter than the others, overreporting of weight was significantly larger. When they recognized the opposite—that they were of greater weight than the others—underreporting of weight became significantly larger. Some relationships differed between boys and girls. In boys, intention to lose weight was significantly associated with underreporting of weight (*p* < 0.01). In girls, questionnaire respondents were significantly associated with misreporting of weight (*p* = 0.03): when girls answered about their weight without their guardians, they underreported it.

**Table 2:** Relationship between misreporting of height/weight and background factors in Japanese primary school children (*n* = 1019)

		Boys (*n* = 486)								Girls (*n* = 533)							
Variable	Category	n	(%)	Misreporting^a^						n	(%)	Misreporting^a^					
				Height (cm)			Weight (kg)					Height (cm)			Weight (kg)		
				mean, SD		*p* value^b^	mean, SD		*p* value^b^			mean, SD		*p* value^b^	mean, SD		*p* value^b^
Age (year)	10	225	(46.3)	-0.17,	2.1	0.49	0.07,	1.5	0.21	233	(43.7)	0.07,	1.2	0.80	-0.02,	1.7	0.10
	11	237	(48.8)	-0.15,	2.4		-0.28,	1.7		267	(50.1)	0.12,	1.8		-0.20,	2.1	
	12	24	(4.9)	0.39,	1.7		0.32,	2.1		33	(6.2)	0.06,	1.3		-0.58,	2.2	
School	A	46	(9.5)	-0.28,	2.5	0.14	-0.07,	1.4	0.08	47	(8.8)	-0.08,	0.8	0.22	-0.10	1.7	0.08
	B	84	(17.3)	0.32,	1.2		0.33,	1.6		87	(16.3)	0.13,	1.5		-0.04	1.5	
	C	77	(15.8)	-0.25,	1.6		-0.21,	1.9		103	(19.3)	0.16,	1.5		0.18	2.2	
	D	101	(20.8)	-0.59,	3.6		0.03,	2.0		103	(19.3)	-0.12,	1.7		0.04	2.0	
	E	37	(7.6)	0.22,	1.5		-0.05,	1.0		24	(4.5)	-0.32,	1.3		-0.17	1.1	
	F	85	(17.5)	0.06,	1.9		-0.42,	1.2		97	(18.2)	0.15,	0.7		-0.64	1.9	
	G	56	(11.5)	-0.22,	1.6		-0.32,	1.6		72	(13.5)	0.43,	2.4		-0.32	2.1	
Measured height (cm)	Small	161	(33.1)	-0.15,	2.5	0.84	0.14,	1.3	0.04	176	(33.0)	0.24,	2.1	0.05	0.12,	1.8	< 0.01
	Medium	160	(32.9)	-0.16,	2.4		-0.19,	1.7		179	(33.6)	0.13,	1.1		-0.10,	1.6	
	Large	165	(34.0)	-0.10,	1.9		-0.22,	1.8		178	(33.4)	-0.09,	1.2		-0.43,	2.2	
Measured weight (kg)	Small	161	(33.1)	-0.32,	2.6	0.27	0.35,	1.5	< 0.01	177	(33.2)	0.38,	2.0	0.02	0.25,	1.5	< 0.01
	Medium	160	(32.9)	-0.04,	2.3		0.08,	1.3		177	(33.2)	-0.09,	1.2		0.06,	1.4	
	Large	165	(34.0)		1.8		-0.68,	1.9		179	(33.6)	-0.01,	1.2		-0.72,	2.5	
Self-awareness of weight	Light	77	(15.8)	-0.37,	3.0	0.08	0.48,	1.8	< 0.01	69	(13.0)	0.69,	2.6	< 0.01	0.36,	1.4	< 0.01
	Adequate	304	(62.6)	-0.18,	2.0		-0.09,	1.4		353	(66.2)	0.02,	1.3		-0.15,	1.9	
	Heavy	105	(21.6)	0.19,	2.2		-0.51,	2.0		111	(20.8)	-0.05,	1.4		-0.43,	2.3	
Intention to lose weight	Yes	113	(23.3)	0.08,	2.6	0.26	-0.53,	1.7	< 0.01	141	(26.5)	0.06,	1.2	0.74	-0.36,	2.0	0.12
	No	373	(76.8)	-0.20,	2.1		0.04,	1.6		392	(73.6)	0.11,	1.6		-0.06,	1.9	
Nutrition knowledge score	Low	156	(32.1)	-0.14,	2.5	0.93	-0.15,	1.8	0.58	165	(31.0)	-0.04,	1.7	0.08	-0.05,	1.9	0.24
	Medium	158	(32.5)	-0.15,	2.3		-0.07,	1.7		182	(34.2)	0.07,	1.3		-0.06,	1.8	
	High	172	(35.4)	-0.12,	2.0		-0.05,	1.4		186	(34.9)	0.24,	1.6		-0.29,	2.0	
Frequency of exercise for > 60 min	0	50	(10.4)	-0.43,	2.2	0.78	0.49,	1.7	0.17	65	(12.4)	0.04,	0.9	0.56	-0.04,	1.9	0.98
(day/1 week)	1 ~ 2	175	(36.3)	-0.01,	2.3		-0.10,	1.5		237	(45.1)	0.10,	1.9		-0.15,	1.7	
	3 ~ 4	136	(28.2)	-0.23,	2.4		-0.41,	1.9		125	(23.8)	0.03,	1.2		-0.34,	2.2	
	5 ~ 7	121	(25.1)	-0.09,	2.1		0.00,	1.4		99	(18.8)	0.22,	1.2		0.02,	2.1	
Questionnaire respondent	Child only	251	(51.7)	-0.19,	2.3	0.39	-0.25,	1.7	0.07	269	(50.5)	0.11,	1.6	0.29	-0.31,	2.2	0.03
	Child + Guardian	169	(34.8)	-0.19,	2.1		0.09,	1.5		210	(39.4)	0.00,	1.3		-0.08,	1.5	
	Guardian only	66	(13.6)	0.22,	2.5		0.08,	1.6		54	(10.1)	0.36,	1.9		0.44,	1.8	

*min* minute, *SD* standard deviation

^a^ Misreporting was defined as the difference between self-reported values and measured values. (Misreporting = self-reported values—measured values)

^b^ Trends of association were examined for ordinary variables using a linear mixed model which assigned scores to the level of the independent variable

To test the difference of average misreporting by schools and by questionnaire respondent, ANOVA was used

The relationship between misreporting of height/weight and BMI z-score is shown in Table 3. Regarding the misreporting of height by girls, the larger their own BMI z-score, the larger their underreporting of height (β: -0.16, 95% confidence interval (CI) [-0.29, -0.03], *p* = 0.02 in Model 3). No significant relationship was observed between misreporting of height and body constitution among boys. To the contrary, as their own BMI z-score increased, underreporting of weight became larger in both boys (β: -0.44, 95%CI [-0.57, -0.30], *p* < 0.01) and girls (β: -0.60, 95%CI [-0.76, -0.45], *p* < 0.01). In addition, in boys only, overreporting of weight was significantly larger when mean BMI z-score in the school was larger (i.e. body constitution of surrounding children in the same school was larger.) (β: 2.57, 95%CI [0.55, 4.59], *p* = 0.01).

**Table 3:** Relationship between misreporting of height/weight and BMI z-score in Japanese primary school children (*n* = 1019)

Dependent variable	Independent variable	Boys (*n* = 486)									Girls (*n* = 533)								
		Model 1^c^			Model 2^c^			Model 3^c^			Model 1^c^			Model 2^c^			Model 3^c^		
		β	95%CI		β	95%CI		β	95%CI		β	95%CI		β	95%CI		β	95%CI	
Misreporting^a^ of	BMI z-score^b^ of individuals	0.19	[-0.01,	0.39]	0.18	[-0.02,	0.38]	0.19	[-0.01,	0.38]	-0.16*	[-0.29,	-0.03]	-0.16*	[-0.29,	-0.03]	-0.16*	[-0.29,	-0.03]
height (cm)	mean BMI z-score^b^ in each school				1.97	[-0.97,	4.92]	2.08	[-1.11,	5.26]				0.68	[-1.13,	2.49]	0.56	[-1.27,	2.38]
	Q respondents Child only							ref									ref		
	Child + Guardian							0.03	[-0.40,	0.47]							-0.10	[-0.38,	0.17]
	Guardian only							0.52	[-0.09,	1.13]							0.27	[-0.17,	0.72]
Misreporting^a^ of	BMI z-score^b^ of individuals	-0.43*	[-0.57,	-0.30]	-0.44*	[-0.58,	-0.30]	-0.44*	[-0.57,	-0.30]	-0.60*	[-0.75,	-0.44]	-0.60*	[-0.75,	-0.44]	-0.60*	[-0.76,	-0.45]
weight (kg)	mean BMI z-score^b^ in each school				2.52*	[0.36,	4.69]	2.57*	[0.55,	4.59]				1.85	[-0.85,	4.56]	2.12	[-0.23,	4.46]
	Q respondents Child only							ref									ref		
	Child + Guardian							0.31*	[0.00,	0.61]							0.23	[-0.10,	0.56]
	Guardian only							0.28	[-0.15,	0.70]							0.80*	[0.27,	1.33]

*BMI* body mass index, *CI* confidence interval, *Q* questionnaire, *SD* standard deviation

*<0.05

^a^ Misreporting was defined as the difference between self-reported values and measured values. (Misreporting = self-reported values—measured values)

^b^ BMI z-score was calculated by a formula based on the body constitution data of Japanese children in 2000

^c^ Multivariate linear mixed models including schools as random effects were used to examine the relationship between the misreporting and body constitution (BMI z-score). As for BMI z-score, coefficient (β) shows the change in misreporting of height or weight when BMI z-score increases 1SD

Effect modification by sex on the relationship between misreporting of height/weight and actual body constitution is shown in Table 4 and Supplementary Fig. 3. Effect modification by sex was significant in the misreporting of height. As BMI z-score increased, ‘overreporting’ of height by boys became significantly larger. In girls, in contrast, as BMI z-score increased, ‘underreporting’ of height became significantly larger. Effect modification in the misreporting of weight was not significant.

**Table 4:** Effect modification by sex on the relationship between misreporting of height/weight and body constitution (*n* = 1019)

Dependent variable	Independent variable	β^a^	95%CI	
Misreporting^b^ of	BMI z-score^c^ of individuals	0.19*	[0.02,	0.36]
height (cm)	Sex	0.17	[-0.07,	0.40]
	BMI z-score x sex	-0.34*	[-0.58,	-0.11]
Misreporting^b^ of	BMI z-score^c^ of individuals	-0.43*	[-0.58,	-0.29]
weight (kg)	Sex	-0.14	[-0.35,	0.08]
	BMI z-score x sex	-0.16	[-0.37,	0.05]

*BMI* body mass index, *CI* confidence interval, *SD* standard deviation

*<0.05

^a^ A multivariate linear mixed model including BMI z-score, sex, and an interaction term (BMI z-score x sex) was used. Schools were also included as a random effect

^b^ Misreporting was defined as the difference between self-reported values and measured values. (Misreporting = self-reported values—measured values)

^c^ BMI z-score was calculated by a formula based on the physical constitution data of Japanese children in 2000

## Discussion

To our knowledge, this is the first study to show the magnitude and direction of height/weight misreporting in Japanese primary school children and its determinants from both individual and environmental perspectives. The height/weight self-reported by children was considered generally accurate among Japanese primary school children, whose height/weight were measured three times a year. Regarding the misreporting of height, the larger the BMI z-score, the larger the ‘overreporting’ of height among boys. Among girls, in contrast, ‘underreporting’ of height became larger with larger BMI z-score. The results were different for the misreporting of weight: children with a larger body size (all of height, weight, and BMI z-score) tended to underreport their weight, irrespective of sex. Misreporting caused by the individual factors could narrow the distribution of self-reported body constitution (BMI), and this may mask relationships between children’s body constitution and other factors. Even when we conduct an epidemiological study in child population, we need to interpret observed results carefully. Regarding environmental factors, boys who belonged to schools with a larger average BMI z-score were more likely to overreport their weight. This tendency was not observed in girls.

In this study, the children’s self-reported height/weight was considered accurate. Yoshitake et al. previously reported the same findings for Japanese children [19]. Seghers et al. reported that the reliability of self-reported height/weight in children was low [31]. However, the frequency of measurement in their study was once every two years, and the participants answered their height/weight without measurement for at least 6 months [31]. In Japan, the School Health and Safety Act mandates that primary schools conduct a health checkup every year, including height/weight measurement [32]. The school year of Japanese primary schools is usually divided into three semesters, and measurement of height/weight is performed at the beginning of every semester. Accordingly, Japanese children likely have greater opportunity to know their own height/weight accurately than children in countries with less rigorous measurement practices.

Before the analysis, we hypothesized that several factors which differ from those in adults may affect misreporting of height/weight by children. Children may not be able to recognize the appropriateness of their own body constitution and/or its positioning within the general population [33]. We therefore suspected that any misreporting of height/weight by children would be unintentional. Regarding adults, past studies suggest that misreporting is largely intentional, and attributable to social norms for body constitution [15, 34, 35]. In our present study, children were asked the question: "Compared to other children in your class, do you think your weight is similar, heavier, or lighter than the others?" to examine their self-awareness of the appropriateness of weight. Comparing their answer to this question with actual body constitution (Supplementary table 1), 38.3% of underweight boys and 52.1% of underweight girls recognized that their weight was adequate or heavy, while 14.0% of normal weight boys and 16.8% of normal weight girls thought that their weight was heavy. A similar discrepancy between actual and self-recognized body constitution was reported in a study of Japanese university students [36], and the authors stated that female students often showed a desire to lose weight even if their body constitution was appropriate. Even in primary school children, some may have the belief that “the thinner, the better,” as is commonly seen in older generations, and some of them may have misreported their height/weight intentionally. This can also cause thinness and its related issues in young Japanese [9–11]. Health education about appropriate body constitution should be provided from the beginning of adolescence.

Given the known relationship between higher nutritional knowledge and healthier eating habits [24], we speculated that higher nutritional knowledge and/or higher frequency of exercise might be related to accurate recognition of height/weight. However, we observed no association between them.

Regarding the misreporting of height by boys, the larger their BMI z-score, the larger their overreporting of height became. This association in boys is consistent with a previous study [37]. In girls, in contrast, the larger their BMI z-score, the larger their underreporting of height. Several previous reports describe the underreporting of height by children, but the results did not differ by sex [38, 39]. A study on the influence of height in the process of mate selection found that the observed frequency of pairings in which women were taller than men was significantly lower than that predicted by a simulation [40]. A male-taller norm appears to exist [40], and tall girls in the present study might have preferred a shorter appearance.

When the body constitution of surrounding children in the same school was larger, only boys tended to overreport their weight. Maximova et al. reported that children perceive themselves to be thinner than their actual BMI when exposed to an obese environment [41]. In our study, the proportion of normal-weight boys who perceived themselves heavier than other classmates was smaller in the schools with a larger mean BMI z-score (10.7%) than in those with a smaller mean BMI z-score (17.7%). It is possible that the size of surrounding people may affect boys’ own weight reporting and their perception of weight adequacy. This relationship was not observed in girls; it is possible that girls’ body image may be more influenced by social norms or information from the media [42] than the body size of relatives and/or children close to them. When parents answered the questionnaires either with or without their children, they tended to overreport their children’s height/weight compared to the replies given by children only. It may be best to ask both children and their guardians to report when the self-reported height/weight of children is used in epidemiological studies.

Our study has several strengths. First, participation rate was relatively high (1019/1231*100 = 82.8%). To minimize selection bias, participants with relatively large misreporting were carefully checked (cutoff value for checking defined based on the average annual growth of height/weight [43]; height [boys ± 13.9 cm, girls ± 11.8 cm], weight [boys ± 9.9 kg, girls ± 9.6 kg]), and as many as possible were included in the analysis. We also performed a sensitivity analysis which excluded those with improbable misreporting, but the results did not substantially change. Second, the measurement of height/weight is standardized by the School Health and Safety Act in Japanese primary schools and performed regularly; thus, the measured height/weight values were accurate. Third, to consider the clustering of participants by the schools, we adopted multivariate linear mixed models which allowed school averages of BMI z-score and individual-level indicators of body constitution to be analyzed simultaneously. Fourth, we asked the children their height/weight just after the health checkup. This enabled us to assess intentional misreporting, because almost all children should have known their actual height/weight. Few previous studies of weight misreporting have had the order of ‘measurement first, survey (self-report) later’.

Several limitations of the study should also be mentioned. First, given that the study was conducted in a single prefecture in Japan, the generalizability of the results should be carefully considered. Nevertheless, the prefecture is located in the central part of the main island of Japan, and includes both urban and rural areas. In addition, the body height/weight of the participants in this prefecture was similar to that in a national survey [43]. Mean height/weight of all children in the surveyed prefecture was 145.6 cm/39.9 kg in boys aged 11 years and 147.4 cm/40.5 kg in girls in 2018 [43]. The same means among all Japanese children of 11 years old were 145.2 cm/38.4 kg in boys and 146.8 cm/39.1 kg in girls. Obvious deviation was not observed between the means of the surveyed area and those of whole Japan. Second, there was no information on growth stage. Since girls generally begin to develop secondary sexual characteristics earlier than boys, the differences seen by sex might be attributable to differences in physical and mental maturity at this age. Third, we do not show the results of adjustments for covariates such as parental education history and socioeconomic states [44], which were reported as confounders in previous studies. Given that this survey was conducted at public schools, it was difficult to ask for the personal information of parents. We did conduct an analysis which included the subjective economic status of the guardian, but the results showed no association with misreporting (not shown in the results). Lastly, although the method of body measurement was standardized, the equipment used at the schools was not unified. In addition, notification timing of the health check-up results to the guardians might have differed between the schools, which might in turn have affected the magnitude of misreporting by the guardians. However, any such differences between schools were considered in multivariate linear mixed models which included school as a random effect.

## Conclusions

Self-reported height/weight was generally accurate in Japanese primary school children. However, even primary school children misreported their height/weight intentionally like adults, possibly due to social pressure to lose weight or that not to stand out. Thus, health education about appropriate body constitution should be provided from the beginning of adolescence, particularly for girls.

## Supplementary Information

**Additional file 1: Supplementary Figure 1.** Agreement between self-reported and measured height/weight for the boys (Bland-Altman plots). Dashed line shows the mean difference between self-reported and measured height/weight. Horizontal lines represent 95% limits of agreement.

**Additional file 2: Supplementary Figure 2.** Agreement between self-reported and measured height/weight for the girls (Bland-Altman plots). Dashed line shows the mean difference between self-reported and measured height/weight. Horizontal lines represent 95% limits of agreement.

**Additional file 3: Supplementary Figure 3.** Association between height/weight misreporting and BMI z-score by SEX (a) Adjusted Predictions of Height misreporting by SEX with 95% CIs (b) Adjusted Predictions of Weight misreporting by SEX with 95% Cis BMI: body mass index ; CI: confidence interval ; SD: standard deviation.

**Additional file 4: Supplementary table 1.** Agreement between self-awareness of weight and measured body constitution (*n*=1019).

## Data Availability

The datasets used and/or analysed during the current study available from the corresponding author on reasonable request.

## Abbreviations

BMI: Body mass index
BDHQ: Brief-type self-administered diet history questionnaire
ANOVA: Analysis of variance
CI: Confidence interval
SD: Standard deviation
