# Supplementary material for: Misreporting of height and weight by primary school children in Japan: a cross-sectional study on individual and environmental determinants
Source: BMC Public Health. 2023 Apr 27;23:775. doi: 10.1186/s12889-023-15682-z (PMC10134671; doi:10.1186/s12889-023-15682-z)
Supplement: Supplementary file 1 — Additional file 1: Supplementary Figure 1. Agreement between self-reported and measured height/weight for the boys (Bland-Altman plots). Dashed line shows the mean difference between self-reported and measured height/weight. Horizontal lines represent 95% limits of agreement. [file 12889_2023_15682_MOESM1_ESM.pdf]

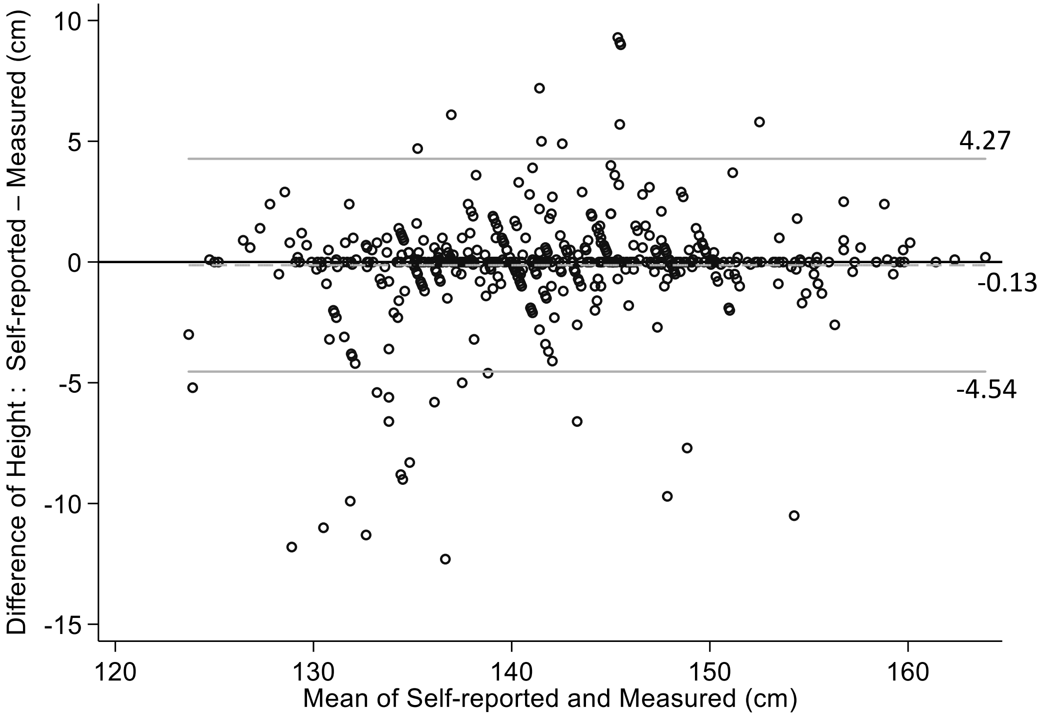

(a) Height

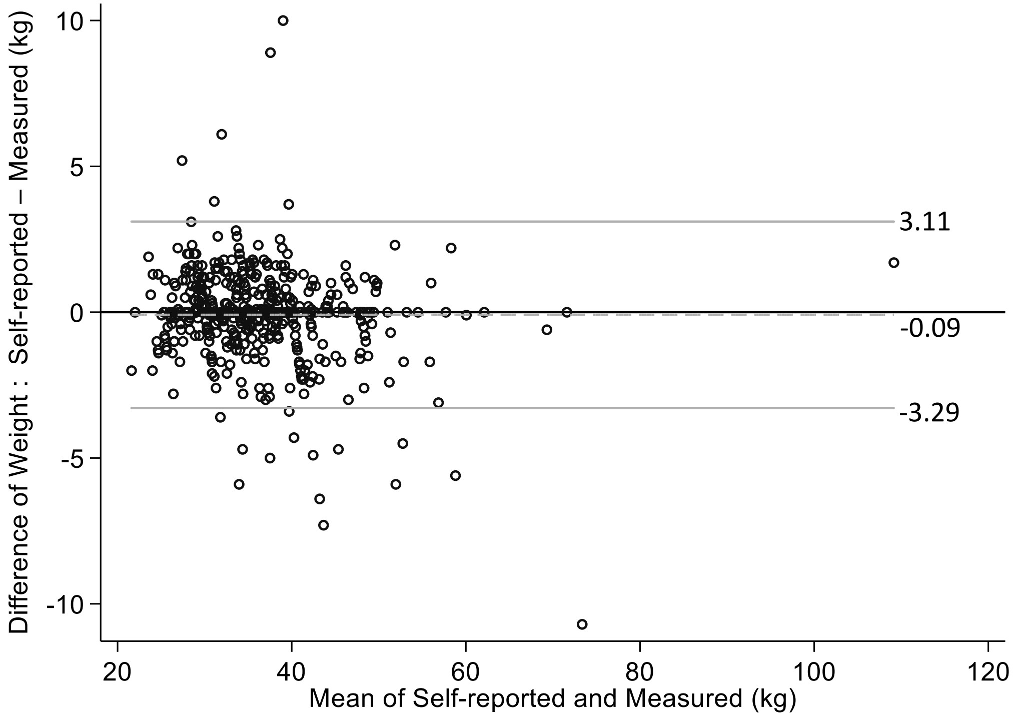

(b) Weight

Supplementary Figure 1. Agreement between self-reported and measured height/weight for the boys (Bland-Altman plots).
Dashed line shows the mean difference between self-reported and measured height/weight. Horizontal lines represent 95% limits of agreement.
