# Supplementary material for: Misreporting of height and weight by primary school children in Japan: a cross-sectional study on individual and environmental determinants
Source: BMC Public Health. 2023 Apr 27;23:775. doi: 10.1186/s12889-023-15682-z (PMC10134671; doi:10.1186/s12889-023-15682-z)
Supplement: Supplementary file 3 — Additional file 3: Supplementary Figure 3. Association between height/weight misreporting and BMI z-score by SEX (a) Adjusted Predictions of Height misreporting by SEX with 95% CIs (b) Adjusted Predictions of Weight misreporting by SEX with 95% Cis BMI: body mass index ; CI: confidence interval ; SD: standard deviation. [file 12889_2023_15682_MOESM3_ESM.pdf]

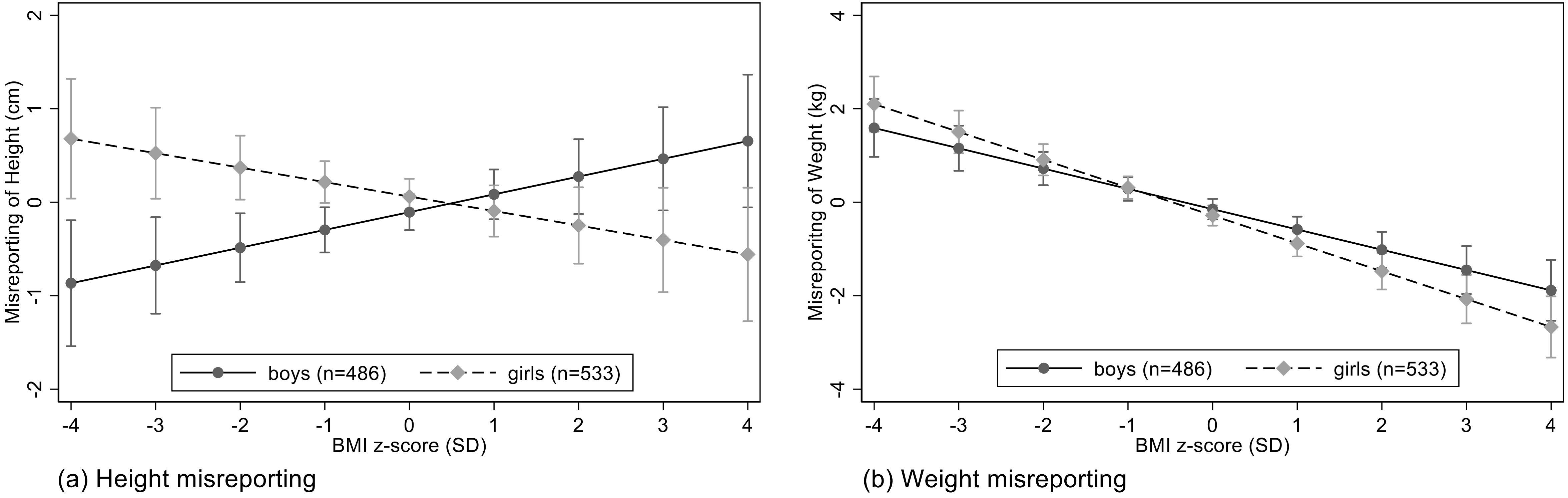

Supplementary Figure 3. Association between height/weight misreporting and BMI z-score by SEX

(a) Adjusted Predictions of Height misreporting by SEX with 95% CIs

(b) Adjusted Predictions of Weight misreporting by SEX with 95% CIs

BMI: body mass index ; CI: confidence interval ; SD: standard deviation
