# Supplementary material for: Misreporting of height and weight by primary school children in Japan: a cross-sectional study on individual and environmental determinants
Source: BMC Public Health. 2023 Apr 27;23:775. doi: 10.1186/s12889-023-15682-z (PMC10134671; doi:10.1186/s12889-023-15682-z)
Supplement: Supplementary file 4 — Additional file 4: Supplementary table 1. Agreement between self-awareness of weight and measured body constitution (n=1019). [file 12889_2023_15682_MOESM4_ESM.pdf]

Supplementary table 1 Agreement between self-awareness of weight and measured body constitution (n=1019)

Sex	Measured body constitution[a]	Self-awareness of weight n(%)			Kappa[b]
		Light	Adequate	Heavy	
Boys (n=486)	Underweight	37 (61.7)	23 (38.3)	0 (0)	0.467 (P<0.001)
	Normal weight	40 (11.2)	267 (74.8)	50 (14.0)	
	Overweight	0 (0)	14 (20.3)	55 (79.7)	
Girls (n=533)	Underweight	34 (47.9)	36 (50.7)	1 (1.4)	0.364 (P<0.001)
	Normal weight	35 (8.6)	302 (74.6)	68 (16.8)	
	Overweight	0 (0)	15 (26.3)	42 (73.7)	

[a] The cutoff values for underweight and overweight were <BMI18.5 and ≥BMI25 at age 18, respectively.

[b] Kappa coefficient was calculated according to Szklo M, Nieto FJ. Epidemiology Beyond the Basics
